# Four Thrombotic Events Over 5 Years, Two Pulmonary Emboli and Two Deep Venous Thrombosis, When Testosterone-HCG Therapy Was Continued Despite Concurrent Anticoagulation in a 55-Year-Old Man With Lupus Anticoagulant

**DOI:** 10.1177/2324709616661833

**Published:** 2016-08-01

**Authors:** Charles J. Glueck, Kevin Lee, Marloe Prince, Vybhav Jetty, Parth Shah, Ping Wang

**Affiliations:** 1 Jewish Hospital of Cincinnati, Cincinnati, OH, USA

**Keywords:** testosterone, human chorionic gonadotropin, Clomid, deep venous thrombosis, pulmonary embolus, anticoagulation

## Abstract

**Background:** When exogenous testosterone or treatments to elevate testosterone (human chorionic gonadotropin [HCG] or Clomid) are prescribed for men who have antecedent thrombophilia, deep venous thrombosis and pulmonary embolism often occur and may recur despite adequate anticoagulation if testosterone therapy is continued. **Case Presentation:** A 55-year-old white male was referred to us because of 4 thrombotic events, 3 despite adequate anticoagulation over a 5-year period. We assessed interactions between thrombophilia, exogenous testosterone therapy, and recurrent thrombosis. In 2009, despite low-normal serum testosterone 334 ng/dL (lower normal limit [LNL] 300 ng/dL), he was given testosterone (TT) cypionate (50 mg/week) and human chorionic gonadotropin (HCG; 500 units/week) for presumed hypogonadism. Ten months later, with supranormal serum T (1385 ng/dL, upper normal limit [UNL] 827 ng/dL) and estradiol (E2) 45 pg/mL (UNL 41 pg/mL), he had a pulmonary embolus (PE) and was then anticoagulated for 2 years (enoxaparin, then warfarin). Four years later, on TT-HCG, he had his first deep venous thrombosis (DVT). TT was stopped and HCG continued; he was anticoagulated (enoxaparin, then warfarin, then apixaban, then fondaparinux). One year after his first DVT, on HCG, still on fondaparinux, he had a second DVT (5/315), was anticoagulated (enoxaparin + warfarin), with a Greenfield filter placed, but 8 days later had a second PE. Thrombophilia testing revealed the lupus anticoagulant. After stopping HCG, and maintained on warfarin, he has been free of further DVT-PE for 9 months. **Conclusion:** When DVT-PE occur on TT or HCG, in the presence of thrombophilia, TT-HCG should be stopped, lest DVT-PE reoccur despite concurrent anticoagulation.

## Case Report

A white male, age 55, was referred to us (June 8, 2015) because of 4 thrombotic events, 3 despite adequate anticoagulation over a 5-year period. We assessed interactions between thrombophilia, exogenous testosterone therapy, and recurrent thrombosis.

A previously healthy 55-year-old skilled welder developed acromegaly and had removal of a growth-hormone secreting pituitary adenoma in 2002. Seven years later (July 2009), after complaints of fatigue and increasing muscle weakness, he was found to have low-normal serum testosterone 334 ng/dL (lower normal limit [LNL] 300 ng/dL) and high estradiol [E2] 45 pg/mL (upper normal limit [UNL] 41 pg/mL), and despite these findings, he was started on 50 mg intramuscular testosterone therapy (TT) cypionate/week and intramuscular human chorionic gonadotropin (HCG) 250 IU twice/week. On TT-HCG, both serum total T and E2 became supranormal (T: 1385 ng/dL, UNL 827 ng/dL; E2: 45 pg/mL, UNL 41).

**Figure 1.**
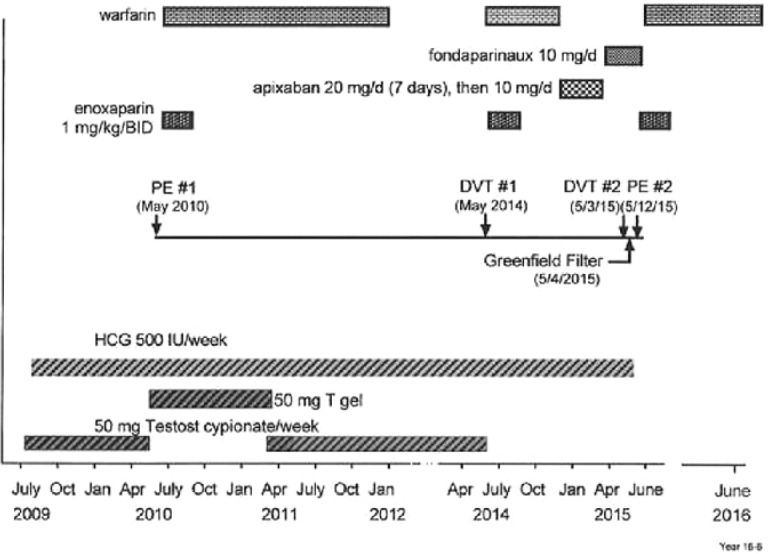
Recurrent pulmonary embolism (PE) and deep venous thrombosis (DVT) despite anticoagulation when testosterone-human chorionic hormone (HCG) therapy was continued in a patient later shown to have the lupus anticoagulant. No further DVT or PE in 9 months after stopping HCG.

Ten months after starting TT-HCG, in May 2010, he sustained multiple pulmonary embolus PE (Figure 1). Sonography for deep venous thrombosis (DVT) was negative, and he was treated with enoxaparin and then warfarin, which was continued for 2 years (Figure 1). At the time of the PE, the injected testosterone cypionate was replaced by testosterone gel 50 mg/day, which was continued along with the HCG for 10 months, after which the gel was changed back to the injections (testosterone cypionate 50 mg/week; (Figure 1).

On TT-HCG (March 18, 2014), his serum E2 was high (54 pg/mL, UNL 41), and total T was normal (543 ng/dL, laboratory normal range 300-827).

In May 2014, 4 years from the initial PE, on TT-HCG but no anticoagulation, DVT #1 in the right leg was diagnosed. TT was stopped, but HCG was continued (Figure 1). Anticoagulation with enoxaparin and warfarin (8 months) was given, then apixaban (20 mg/day for 7 days, then 5 mg BID) and then fondaparinux 10 mg/day for another 4 months (Figure 1).

On May 3, 2015, 1 year after his first DVT, despite having been on enoxaparin, then warfarin, then apixaban, and then fondaparinux, while still on HCG, his second DVT occurred (Figure 1). Venous Doppler revealed a 20″ long nonocclusive thrombus in the right leg. A Greenfield filter was placed (May 4, 2015), enoxaparin and warfarin were re-instituted, and the HCG was stopped on our consultation (May 10, 2015). However, his second PE occurred 8 days after placement of the Greenfield filter (Figure 1) while still receiving both enoxaparin and warfarin (Figure 1). After stopping the HCG, and maintained on enoxaparin and warfarin together, and then warfarin alone for 9 months, he has had no recurrent DVT-PE (Figure 1).

We evaluated the patient for the first time in Cincinnati on May 28, 2015. Five years from his first PE, our tests of thrombophilia and hypofibrinolysis revealed the presence of the lupus anticoagulant, measured while on warfarin, with DRVVT 57.9 seconds (reference <55) and hexagonal phospholipid neutral 14.

Being off the HCG for 2 weeks and off TT for a year, serum T was normal (622 ng/dL; laboratory normal range 348-1197 ng/dL), and E2 was normal (19.1 pg/mL; laboratory normal range 7.6-42.6 pg/mL).

## Discussion

TT is often indiscriminately prescribed^1^ without consideration of risks.^2^ As in this case, many men given TT do not meet well-defined diagnostic and therapeutic criteria.^3^ Before TT-HCG, our patient was found to have low-normal serum T (334 ng/dL; LNL 300 ng/dL) and high E2 (45 pg/mL, UNL 41 pg/mL). Despite these findings, he was started on 50 mg testosterone cypionate and HCG 500 IU per week. On TT-HCG serum total T became supranormal (1385 ng/dL, UNL 827), and serum E2 was high (45 pg/mL; UNL 41), both speculatively contributing to his DVT-PE events, interacting with his lupus anticoagulant.

In 36 hypogonadal patients in our center studied before and on conventional TT (50-60 mg gel/day) 11% developed supranormal serum T (>800 ng/mL) on TT.^4^ In these 36 patients, E2 was supranormal (>42.6 pg/mL) before TT in 6%, and on TT in 14%. Conventional TT can lead to supranormal T and E2 in hypogonadal patients.

We have previously described 11 of 67 patients with thrombophilia given exogenous TT^5,6^ who sustained a first venous thromboembolic event (VTE) and then, while continuing TT, and despite adequate anticoagulation, sustained a second VTE. Moreover, of these 11 patients, with TT further continued, 6 men^4-6^ had a third VTE, despite continuing adequate anticoagulation. Recurrent PEs and DVTs despite anticoagulation in our current case when TT-HCG or HCG alone were continued parallels our previous 11 patients’^4-6^ recurrent thrombotic events. After stopping HCG, with continuation of warfarin, in the current study, the subject had no further DVT-PE over the subsequent 9 months of follow-up.

In the current case, the sole thrombophilia identified was the lupus anticoagulant, which was present during warfarin anticoagulation. Recent studies have suggested that despite concurrent warfarin, the lupus anticoagulant can be detected by dilute Russel’s Viper Venom time.^7,8^ However, the lupus anticoagulant can be transient and should be remeasured over time for optimal confirmation.

In 2014, both the US Food and Drug Administration^9^ and Canada Health,^10^ based on postmarketing data, required manufacturers to add a warning to testosterone product labels about the potential risks of VTE, including DVT and PE. As a result, there is now broad public concern regarding this issue.

TT and/or HCG are conventionally used in the treatment of hypogonadism.^11^ High serum E2 is common during TT therapy^5,6,12,13^ via aromatization from exogenous T and may provide a direct stimulus to thrombosis, particularly when, as in the current case, TT supplementation leads to supranormal serum T, and subsequently to supranormal E2. TT can also promote VTE via increased blood pressure,^14^ polycythemia,^15,16^ decrements in high-density lipoprotein cholesterol,^14,16^ blood hyperviscosity, and platelet aggregation.^16-18^ Intramuscular TT increases platelet thromboxane A2 receptor density and platelet aggregation,^19^ increasing adhesion to the coronary artery endothelium and thrombus formation, with subsequent plaque rupture and acute coronary syndrome.^20^ Dihydrotestosterone can promote acute coronary events through enhanced monocyte activation.^21,22^

From 2001 to 2011, androgen use in men age ≥40 has increased more than 3-fold.^23^ Despite the example of thrombotic and cardiovascular events related to sex-hormone therapy in postmenopausal women from the Women’s Health Initiative,^24,25^ TT is rapidly increasing, often indiscriminately, without understanding of its long-term effects.^1,26^

Use of TT in young men with classic forms of hypogonadism is considered effective and there is little disagreement regarding its use in this patient population.^3^ However, T levels fall with age,^27,28^ with chronic disease,^29,30^ and with obesity^29^ but not with smoking.^31,32^ With aging, accumulation of obesity, and development of diabetes, more and more men have lower T, but do not meet diagnostic criteria^3^ for hypogonadism. Moreover, most normal ranges come from healthy younger men.^33^ Differences in T assay methods^34,35^ and adverse muscle symptoms at different T levels further interfere with efficient diagnosis.^36^

Use of TT in men affected by late onset hypogonadism (LOH),^37,38^ where low T has no definable etiology beyond aging and/or chronic disease, is controversial, with concerns over its effectiveness and safety.^2,39-42^ Indiscriminate use of TT for LOH is a real issue,^2,4,5,43-45^ as this form of therapy is administered to almost 1 in 25 American men over the age of 60.^46^

Increasing use of TT may have thrombotic^12,13,47-50^ and cardiovascular ramifications.^2,51,52^ Adverse or intermediate cardiovascular disease (CVD) outcomes have been widely reported after TT was started.^2,14,16,41-43,51,53,54^ In contrast, 2 studies demonstrated significant CVD event reduction on TT.^39,40^ A placebo-controlled clinical trial demonstrated that in patients age 65 or older, raising low testosterone to mid-normal range improved sexual function, mood, and symptoms of depression, but failed to improve vitality and walking distance.^55^ The sample size was too small provide conclusions about TT risks.^55^

We have compared 67 patients who sustained VTE^4^ after starting TT to 76 controls, not taking TT, who sustained DVT/PE. Of the 67 VTE TT patients, 16 (24%) were found to be heterozygous for the factor V Leiden mutation versus 7 of the 76 (7%) VTE controls not on TT, *P* = .004. The 67 patients with VTE on TT also were more likely than the 76 VTE no-TT controls to have the lupus anticoagulant, 9/64 (14%) versus 2/76, 3%, *P* = .023.^4^ When screening men^4^ for thrombophilia before starting TT, we suggest that minimal tests include the factor V Leiden mutation and the lupus anticoagulant, while more extensive tests would also include PCR for the G20210A prothrombin gene mutation, factors VIII and XI, and homocysteine.

In contrast to our clinical case-control series,^4^ in an observational population study by Baillargeon et al,^56^ TT use was not associated with increased VTE events. Having filled a prescription for TT was not significantly associated with increased risk of VTE.^56^ In a retrospective study of male adults with low serum T at a low-moderate baseline risk of DVT/PE, and after excluding men with prior history of DVT/PE, cancer, hypercoagulable state, and chronic anticoagulation, Sharma et al^57^ did not detect a significant association between TT and risk of DVT/PE. However, almost all of our 67 subjects^4^ with VTE after starting TT would have been excluded by Sharma et al^57^ because of antecedent DVT/PE, hypercoagulable state, and chronic anticoagulation, biasing against recognizing a significant association^4^ between TT and DVT-PE events.^57^

Particularly since our recent retrospective study of men and women hospitalized for VTE^44^ revealed that personal and family history were nonspecific and insensitive to effectively identify subjects in whom TT and estrogen replacement therapy should not be given, prescreening for thrombophilia is realistic. However, it is expensive, with average laboratory costs in our center $1200. On the other hand, direct hospital costs for VTE events are about $15 000,^58^ and higher for re-admission, and there are high direct and indirect costs of post–phlebitic syndrome. It would be valuable to do a pharmacoeconomic study of the number of quality of life years realized by prescreening for coagulation factors before starting TT, and the balance between the cost of hospitalization for VTE and dollars saved by prescreening. This analysis should provide an incremental cost-effectiveness ratio within a society willingness-to-pay threshold.

Why TT induces VTE in thrombophilic patients despite correct use of anticoagulation is unknown, but we speculate that testosterone with subsequent aromatization to estradiol must interact with underlying coagulation factors to overwhelm the expected protection from the anticoagulant.

A limitation of our study is that there is only one set of lupus anticoagulant data. The lupus anticoagulant may be transient,^59^ and therefore we do not know that it was present during the entire time frame that the patient was studied. Even if the lupus anticoagulant was only transient, based on this case and in 11 similar cases (of 67 previously reported),^4^ we would never continue TT despite concurrent anticoagulants in patients with previous VTE, particularly if they had familial thrombophilia or repetitively demonstrated lupus anticoagulant.

## Conclusions

When DVT-PE occurs in men or women^48^ given exogenous TT and/or HCG, and especially in the presence of thrombophilia-hypofibrinolysis,^4-6^ we believe that continued or subsequent use of TT is contraindicated. As in this case, and in the presence of thrombophilia, continuation of anticoagulation therapy during TT repetitively fails to prevent recurrent DVT-PE, and to date, the only solution is to stop the TT. Moreover, we suggest that screening for thrombophilia be done before starting TT in order to avoid interaction of TT with underlying thrombophilia with resultant VTE. If thrombophilia is found before starting TT, this substantially changes estimations of risk-benefit ratios related to TT.
