# CORRIGENDUM to Four Thrombotic Events Over 5 Years, Two Pulmonary Emboli and Two Deep Venous Thrombosis, When Testosterone-HCG Therapy Was Continued Despite Concurrent Anticoagulation in a 55-Year-Old Man With Lupus Anticoagulant

**DOI:** 10.1177/2324709616689401

**Published:** 2017-01-01

## Abstract

Owing to errors made by the authors, Charles J. Glueck, Kevin Lee, Marloe Prince, Vybhav Jetty, Parth Shah, and Ping Wang, the following article contains errors.

Glueck CJ, Lee K, Prince M, et al. Four Thrombotic Events Over 5 Years, Two Pulmonary Emboli and Two Deep Venous Thrombosis, When Testosterone-HCG Therapy Was Continued Despite Concurrent Anticoagulation in a 55-Year-Old Man With Lupus Anticoagulant. *J Investig Med High Impact Case Rep.* 2016;4(3):1-6. doi: 10.1177/2324709616661833

The following correction applies:

The fifth author’s name should have been mentioned as “Parth Shah” instead of “Parth Shah, MD”. The correct author list is given below.

Charles J. Glueck, MD, Kevin Lee, MD, Marloe Prince, MD, Vybhav Jetty, MD, Parth Shah, and Ping Wang, PhD.

